# The Aberrometric Effect of Corneal Plus Power Ring Distribution on Axial Length Growth in Myopic Children Undergoing Orthokeratology Treatment

**DOI:** 10.3390/children13010025

**Published:** 2025-12-23

**Authors:** Ana Maria Espín, Lluisa Quevedo, Jaume Pauné, António Queirós

**Affiliations:** 1 Faculty Optics and Optometry of Terrassa, Barcelona Tech (UPC), 08034 Barcelona, Spain; luisa.quevedo@upc.edu; 2 Clinical and Experimental Optometry Research Lab (CEORLab), School of Science, University of Minho, 4710-057 Braga, Portugal; aqp@fisica.uminho.pt; 3 Physics Center of Minho and Porto Universities, University of Minho, 4710-057 Braga, Portugal

**Keywords:** orthokeratology, back optic zone diameter (BOZD), plus power ring diameter (PPRD), pupil diameter, aberrations, axial length

## Abstract

Background/Objectives: Myopia progression is strongly associated with axial length (AL) elongation, and orthokeratology (Ortho-K) lens design may influence treatment outcomes. This study has the aim to evaluate the impact of lens customization as optical zone diameter between specific higher-order aberrations (HOA) and axial length (AL) changes in myopic children. Methods: This retrospective study evaluated 66 Caucasian myopic children (mean age, 13.3 ± 1.4 years, 60% male) fitted with Ortho-K lenses with varying back optic zone diameters (BOZD, 4.7–6.0 mm) in a Spanish optometric clinic. Baseline mean spherical equivalent (sphere + 1/2 cylinder) was −2.94 ± 1.24 D and AL = 24.52 ± 0.80 mm. Results: After 12 months, children fitted with smaller BOZDs showed significantly less axial elongation than those with larger BOZDs (0.08 ± 0.12 mm vs. 0.15 ± 0.10 mm, *p* < 0.001) and smaller plus power ring diameter (PPRD). Differences in AL change were observed between PPRD subgroups (larger and smaller than 4.5 mm). HOA revealed distinct patterns: vertical coma increased significantly only in the PPRD > 4.5 mm group (*p* = 0.003), horizontal coma increased significantly only in the PPRD < 4.5 mm group (*p* = 0.004), while total coma increased in both, without intergroup differences. Both PPRD subgroups demonstrated significant increases in spherical aberration (*p* < 0.001). Conclusions: These findings suggest that reducing BOZD, and consequently PPRD, can slow AL elongation more effectively than standard designs, although optical side effects require consideration. Further studies should clarify the interplay of BOZD, PPRD, and pupil size in myopia control.

## 1. Introduction

Myopia is the most prevalent refractive error and one of the main causes of visual impairment in the world [1,2,3], with a significant increase in recent years. In fact, the prevalence of myopia is expected to reach 50% of the world’s population by 2050 [4], of which one-fifth will develop high myopia [5,6] highlighting the urgent need to develop effective prevention and control strategies [7,8].

To control and slow the progression of myopia, a variety of optical interventions have been explored, including ophthalmic lenses and daytime myopia control contact lenses. One of the emerging strategies for controlling myopia progression is orthokeratology (Ortho-K), which has shown promise in clinical practice [9].

Ortho-K is a treatment that uses rigid gas permeable contact lenses with reverse geometry, designed to be worn at night, shaping the cornea and providing clear vision during the day [10,11]. This treatment is reversible and has been identified as effective in reducing the progression of myopia in children and adolescents, as well as in improving daytime visual acuity without the need for optical correction and, in particular, controlling the increase in axial length [12,13].

Several studies have shown that Ortho-K is a safe and effective option for myopia control, with effects that may persist even after lens removal, although continued use is required to maintain the benefits [14].

The exact mechanism of how Ortho-K lenses slow myopic progression is still under investigation, although the most widely accepted theory is that of peripheral defocus. The lenses create myopic blur at the periphery of the retina, which is thought to reduce the stimulus for axial lengthening of the eye [15,16].

The axial length of the eye is one of the main determinants of ocular change, and its increase is directly associated with the development of progressive myopia. Recent research has indicated that controlling axial length through treatments such as Ortho-K could be a key strategy to slow the progression of myopia in the long term [17].

However, this treatment also induces changes in optical aberrations [18,19], playing an important role in myopia and its progression [20]. Optical aberrations were distortions in the image formed in the retina, and they can be of the first and second order, classified as myopia, hyperopia and astigmatism, or of higher order, such as coma or spherical and oblique astigmatism. Zernike’s coefficients [21] were key tools to characterize these distortions in the image. Ortho-K produces changes in corneal aberrations, especially in high-order aberrations, such as spherical aberration and coma, which can affect the quality of vision [22].

Recently, it has been suggested that correction of higher-order aberrations through advanced technologies in contact lenses or refractive surgery could improve visual quality in myopic people, which in turn could have an impact on myopia control [19]. Specifically, the control of optical aberrations through Ortho-K could be beneficial not only in terms of visual improvement but also in reducing the progression of myopia, since it has been observed that the change in the shape of the cornea can influence the modulation of ocular aberrations [23].

In addition, pupil size plays a crucial role in the efficacy of treatment, as a larger pupillary diameter can improve the effectiveness of peripheral myopia correction [24] and simultaneously increase the optical aberrations in the eye.

Studies have also pointed out that customization of lens design, such as variation in optical zone diameter, can influence treatment outcomes, showing that a smaller diameter may be more effective in reducing axial length in certain cases [25,26]. Its relationship with optical aberrations change and its impact on axial length growth require more studies to reach definitive conclusions. Controlling myopia through this treatment remains an active area of research, with an emphasis on improving lens designs and better understanding of the physiological mechanisms involved. This study has the aim to evaluate the impact of lens customization as optical zone diameter and its relationship between specific HOA, such as horizontal coma, vertical coma, total and spherical aberrations, and axial length changes in myopic children.

## 2. Materials and Methods

### 2.1. Study Design and Clinical Population

This is a retrospective study conducted in accordance with the tenets of the Declaration of Helsinki and which was approved by the Ethics Committee Review Board of the Teknon Medical Center. Dataset of 500 records of patients aged 7 to 14 years with axial length data recorded both, at baseline and after one year of Ortho-k treatment, who consulted the Myopia Control Unity of the Teknon Medical Center in Barcelona, between March 2012 and October 2016 was considered. For the present study, a total of 66 subjects treated with Ortho-K lenses with different Back Optic Zone Diameter (BOZD) (range, 4.7–6.0 mm) were included.

Subjects were selected based on topographic maps showing a centered treatment zone (within 0.5 mm of the visual axis) and a uniform treatment zone (TZ). Eligible patients had myopia ranging from −0.50 D to −5.00 D, with astigmatism less than −1.00 D, and achieved successful treatment outcomes with a residual refractive error of ≤0.50 D. Only participants who completed all scheduled Ortho-k follow-up visits (initial visit; after 1 night; at 1 week; 1 month; 3 months; 6 months; and 12 months) were considered. Consequently, subjects with incomplete data or missed visits were excluded from the analysis to ensure the integrity of the longitudinal follow-up.

### 2.2. Orthokeratology Lens Design

Participants were fitted with a Double Reservoir Lens design. These lens design incorporates a secondary lacrimal reservoir that enhances hydrodynamic suction and pressure forces, resulting in improved centration and producing the fastest epithelial changes. All lens parameters were optimized to achieve proper centration and refractive outcomes. Depending on the patient’s corneal characteristics, spherical or toric designs were selected to ensure optimal treatment performance.

Lenses were manufactured from Boston XO (hexafocon A), a material with oxygen permeability of 100 ISO/Fatt, refractive index of 1.415, Rockwell R hardness of 112 units, and a wetting angle of 49 degrees measured with the captive bubble method.

The reduction in the BOZD was achieved by widening the peripheral curves while maintaining a constant total lens diameter and sagittal height. The curvature of the reverse curve was pre-adjusted by the manufacturer to ensure that the lens fit remains consistent for a given corneal profile.

### 2.3. Procedures

All participants underwent an optometric examination prior to the Ortho-K lens fitting. This included a thorough slit-lamp evaluation of the cornea and ocular adnexa, measurement of uncorrected monocular and binocular visual acuity, a refractive examination, measurement of corrected monocular and binocular visual acuity, corneal topography, assessment of corneal and ocular aberrations, and axial length measurement.

Patients underwent four corneal topographies, which were used to calculate and manufacture customized Ortho-k lenses according to the manufacturer’s protocol, based on topographic curvature values and the subject’s refractive error. Once the lenses were manufactured, an initial fitting visit was conducted to ensure proper lens-to-cornea alignment. Patients were instructed on lens handling, as well as on cleaning and maintenance procedures.

At all follow-up visits—after one night, one week, and at 1, 3, 6, and 12 months of lens wear—the following assessments were performed: slit-lamp examination of the cornea and adnexa, measurement of uncorrected and corrected monocular and binocular distance visual acuity, refractive assessment, corneal topography, corneal and ocular aberration measurements, and axial length (AL) evaluation.

Axial length was measured using the IOL Master 500 (Cal Zeiss, Jena, Germany), after one month of treatment and again after 12 months of orthokeratology wear to assess longitudinal changes.

**Figure 1:**
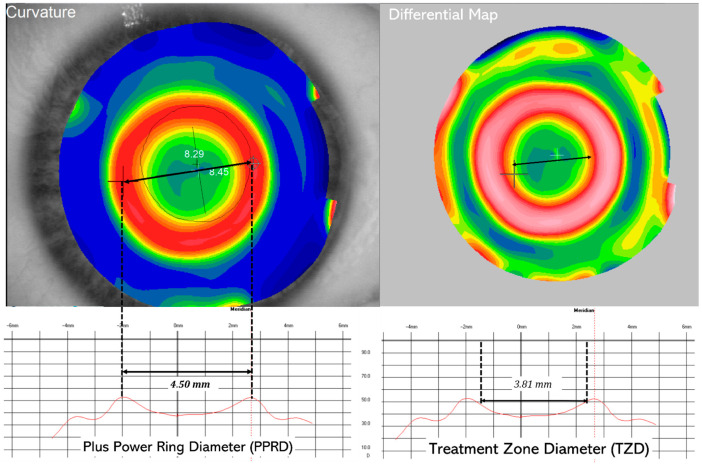
On the left a Post treatment Tangential Curvature map, in mm, showing the effect of Ortho-K lens on a cornea and graphical representation of plus power ring diameter (PPRD, mm). Hotter colors (red-orange) identify the location for the minimum radius of curvature (corneal steepening). On right image a differential map (post-pre) showing the treatment zone diameter in the same patient.

Tangential topographic maps were retrieved before Ortho-K using the Keratron Onda topographer-aberrometer (Keratron, Rome, Italy). The points of relatively higher plus power change (steeper curvature radius) within the PPR were identified at baseline and after 12 months of treatment. The PPR diameters (PPRD) along the horizontal and vertical meridians were then determined, and the median value was calculated as seen in Figure 1. The cut-off point of 4.5 mm for PPRD was selected based on the median value of the PPRD distribution in the study population, which allowed dividing participants into two groups (PPRD < 4.5 mm and PPRD ≥ 4.5 mm) with an equal number of eyes per group (*n* = 33 each). This data-driven approach ensured a balanced sample size and avoided bias associated with arbitrary thresholds. As no previous clinical standard for PPRD cut-off values has been established in the literature, using the median provided an objective criterion for group allocation and allowed an unbiased statistical comparison of the relationship between PPRD and axial length change.

Aberration measurement protocol**.** Ocular aberrations were measured with the Keratron Onda topographer–aberrometer (Optikon, Rome, Italy), which uses a Hartmann–Shack wavefront sensing system. The aberration data were decomposed into Zernike polynomials up to the sixth order, and results were expressed as root mean square (RMS) values. All aberration measurements were performed without cycloplegia. Assessments were performed under scotopic conditions, with the contralateral eye occluded and the patient fixating on the internal target to minimize accommodation. A fixed pupil diameter of 4.0 mm was used for analysis across all subjects to ensure comparability between pre- and post-treatment values.

All Ortho-K fittings and evaluations were performed by a single expert examiner. Data were collected after refractive and topographic stabilization was achieved. Only the right eye of each patient was considered for statistical analysis.

### 2.4. Statistical Analysis

Statistical analysis was performed using SPSS v23.0 (IBM Inc., Chicago, IL, USA). The normality of data distribution was assessed using the Shapiro–Wilk test. Depending on the data distribution, either the independent samples *t*-test or the Mann–Whitney U test was applied to compare baseline variables between groups in the RPRD comparison. For within-group comparisons (pre- and post-treatment), the paired samples *t*-test or the Wilcoxon signed-rank test was used, depending on whether the variables were normally or non-normally distributed, respectively. Given that the main outcomes of the study rely on two primary between-group comparisons, the significance threshold for these analyses was adjusted using the Bonferroni correction (α = 0.025). In addition to null-hypothesis significance testing, effect sizes were calculated to quantify the magnitude of observed differences. For between-group comparisons of continuous variables we computed Cohen’s d (using pooled standard deviation) and interpreted effect sizes according to conventional thresholds (small ≈ 0.2, medium ≈ 0.5, large ≈ 0.8). For within-group (pre–post) comparisons, standardized mean change (mean difference divided by pooled SD) was used where appropriate. Differences were considered statistically significant when the *p*-value was less than 0.05.

## 3. Results

A total of 66 eyes were included in the study. To better assess the annual AL elongation in myopic children, subjects were divided into two groups based on the mean of the PPRD value as a cut-off: The PPRD < 4.5 group (*n* = 33) and the PPRD ≥ 4.5 group (*n* = 33). The comparative results between both groups are presented in Table 1.

At baseline, the myopia ranged from −0.50 to −6.00 D (mean, −2.94 ± 1.24 D in the group PPRD < 4.5 and −3.12 ± 1.52 in PPRD ≥ 4.5). No statistically significant differences were observed in baseline Mean Spherical Equivalent refraction (Sphere + 1/2 Cylinder) (M; *p* = 0.301). A Student’s *t*-test was performed, showing that there were no significant changes in refraction before and after treatment in either group (mean, −0.35 ± 0.43 D in the group PPRD < 4.5 and −0.48 ± 0.41 in PPRD ≥ 4.5, difference 0.13 ± 0.10 D, *p* = 0.102).

### 3.1. Pupil Size

Similarly, the photopic pupil diameter (Pupil Photopic) was slightly larger in the PPRD < 4.5 group (4.34 ± 0.63 mm) compared to the PPRD ≥ 4.5 group (4.14 ± 0.53 mm), although this difference did not reach statistical significance (*p* = 0.084).

However, a statistically significant difference was found in the scotopic pupil diameter (Scotopic Pupil), which was greater in the PPRD < 4.5 group (6.58 ± 0.80 mm) than in the PPRD ≥ 4.5 group (5.95 ± 1.01 mm), with a mean difference of 0.63 ± 0.22 mm (*p* = 0.003).

We investigated the relationship between photopic and scotopic pupil size, as well as mean PPRD and axial length (AL) change after 1 year. Correlation analyses showed that neither photopic nor scotopic pupil size was significantly associated with AL change (photopic: r = −0.05, *p* = 0.722; scotopic: r = −0.03, *p* = 0.820). Similarly, correlations between pupil size and mean PPRD were non-significant for photopic measurements (r = −0.208, *p* = 0.093), whereas scotopic measurements showed a moderate negative correlation (r = −0.40, *p* < 0.001). Finally, AL change and mean PPRD were positively correlated (r = 0.40, *p* < 0.001).

### 3.2. PPRD and BOZD

As expected, based on the classification criteria, the mean PPRD value was significantly higher in the PPRD ≥ 4.5 group (5.12 ± 0.39 mm) compared to the PPRD < 4.5 group (4.06 ± 0.26 mm), with a mean difference of −1.06 ± 0.08 mm (*p* < 0.001).

Similarly, a significant difference was observed in the back optic zone diameter (BOZD), with values of 6.02 ± 0.50 mm for the PPRD ≥ 4.5 group and 5.01 ± 0.34 mm for the PPRD < 4.5 group (*p* < 0.001). BOZD correlated with PPRD with a R^2^ of 0.67 (*p* < 0.001), meaning that a reduced BOZD induced a smaller treatment zone diameter.

### 3.3. Axial Length

**Figure 2:**
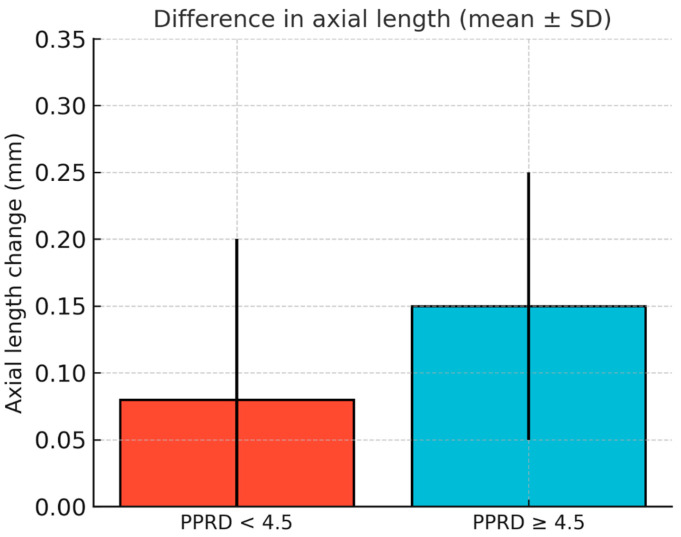
Distribution of AL change according to PPRD in the two groups before and after orthokeratology treatment. SD are shown as bars.

Regarding AL, neither the baseline nor the follow-up measurements showed statistically significant differences between groups (AL Baseline: *p* = 0.158; AL post: *p* = 0.092), although a trend toward higher values was identified in the PPRD ≥4.5 group. However, the change in axial length at one year was significantly greater in this group (0.15 ± 0.10 mm) compared to the PPRD < 4.5 group (0.08 ± 0.12 mm), with a difference of 0.07 ± 0.03 mm (*p* = 0.008) between groups, as seen in Figure 2.

The difference in AL change at 1 year between the groups (PPRD < 4.5: 0.08 ± 0.12 mm vs. PPRD ≥ 4.5: 0.15 ± 0.10 mm) was statistically significant (*p* = 0.008). The between-group effect size for this comparison was Cohen’s d = 0.63, indicating a moderate magnitude. For reference, the difference in mean PPRD (4.06 ± 0.26 mm vs. 5.12 ± 0.39 mm) and BOZD (5.01 ± 0.34 mm vs. 6.02 ± 0.50 mm) yielded very large effect sizes (d = 3.20 and d = 2.36, respectively), which reflect the grouping criterion rather than an independent treatment effect. The difference in scotopic pupil diameter (6.58 ± 0.80 mm vs. 5.95 ± 1.01 mm; *p* = 0.003) showed a moderate-to-large effect (d = 0.69). These results suggest that smaller PPRD and BOZD values are moderately associated with lower axial elongation over one year of orthokeratology wear.

A multiple linear regression model was conducted to evaluate the influence of baseline refraction (M baseline), photopic pupil size, scotopic pupil size, and mean PPRD on axial length (AL) change after one year. The overall model explained approximately 19% of the variance in AL change (R^2^ = 0.190, *p* = 0.011).

Among the predictors, mean RPRD emerged as the only statistically significant factor (β = 0.086, *p* < 0.001). Neither M baseline (β = 0.008, *p* = 0.44), photopic pupil size (β = 0.006, *p* = 0.82), nor scotopic pupil size (β = 0.015, *p* = 0.38) showed significant associations with AL change.

Standardized regression coefficients highlighted the relative weight of each variable. Mean RPRD had the largest standardized effect (β = 0.53), whereas scotopic pupil (β = 0.14), M baseline (β = 0.11), and photopic pupil size (β = 0.03) contributed minimally.

### 3.4. Higher-Order Aberrations

Pre and post treatment changes in wavefront aberrations were analyzed, comparing groups with PPRD < 4.5 and ≥ 4.5. Variables evaluated included vertical coma (VC), horizontal coma (HC), Coma RMS (Coma), and Spherical Aberration RMS (SA) (Table 2).

Regarding vertical coma (VC), the PPRD ≥ 4.5 group showed a significant increase in VC after treatment (Δ = 0.084 ± 0.160 μm; *p* = 0.003), while no changes were observed in the PPRD < 4.5 group (*p* = 0.475). The intergroup difference in change was also significant (0.086 ± 0.160 μm; *p* = 0.021), indicating that subjects with larger PPRD experience greater change towards positive value for vertical coma after treatment.

Regarding horizontal coma (HC), the PPRD < 4.5 group showed a significant positive value increase, changing from close to zero (−0.004 ± 0.060 μm) to positive (0.071 ± 0.153 μm; *p* = 0.004), whereas in the ≥4.5 group, the change was not significant (*p* = 0.061). However, comparison of the change between groups revealed a significant difference (ΔHC: −0.134 ± 0.044 μm; *p* = 0.004), indicating greater differences in this aberration in groups, where eyes with a lower PPRD trended to positive, while eyes with a larger PPRD trended to negative HC post-treatment.

For Coma RMS (C), both groups showed a significant increase after adaptation (group < 4.5: Δ = 0.136 ± 0.159 μm, *p* < 0.001; group ≥ 4.5: Δ = 0.118 ± 0.218 μm, *p* = 0.002), with no significant difference between the two groups (*p* = 0.353).

**Figure 3:**
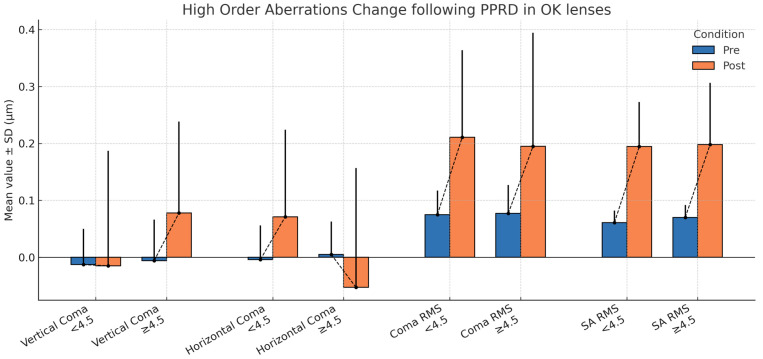
Distribution of aberrations behavior in the two groups, PPRD > 4.5 and PPRD < 4.5 before and after orthokeratology treatment. Values in microns.

An increase in SA RMS was observed after treatment in both groups (group < 4.5: Δ = 0.134 ± 0.080 μm; group ≥ 4.5: Δ = 0.128 ± 0.112 μm), with this increase being significant in both cases (*p* < 0.001). The difference between the two groups was not significant (*p* = 0.440). A visualization of these findings and trends may be seen in Figure 3.

## 4. Discussion

The findings of this study confirm that customizing an Ortho-K lens to achieve a reduced treatment zone diameter [27] significantly influences the modulation of axial elongation in myopic children. Reduction in BOZD induces changes in the called plus power ring diameter [PPRD] and in the treatment zone diameter [28], hence in the treatment zone diameter. In particular, following our results, a PPRD smaller than 4.5 mm was associated with a lower annual increase in axial length compared to a PPRD equal to or greater than this value. This finding seems to support the hypothesis that a greater proportion of myopic defocus induced within the pupillary area may be a key mechanism in slowing myopia progression [29,30,31], as defocus in the region closest to the macula can create an optical signal that slows down axial growth in myopic children [32]. Nevertheless, another explanation may be that increased HOAs, as well as intraocular scatter, can reduce retinal image quality and thereby influence treatment outcomes.

These findings indicate that axial elongation at one year is predominantly influenced by mean RPRD, while baseline refractive error and pupil size parameters play a negligible role in predicting AL progression in this cohort, as seen in Figure 2.

Results of this study are also consistent with previous research indicating that customized lens designs, such as modifications in the back optic zone diameter [BOZD] [33,34], can have substantial effects on Ortho-K treatment outcomes [28,35]. It is important to emphasize the distinction between BOZD and PPRD when interpreting these results. The Back Optic Zone Diameter (BOZD) is a lens design parameter chosen by the clinician and can be intentionally modified to influence the corneal reshaping pattern. In contrast, the Plus Power Ring Diameter (PPRD) is a measured characteristic of the corneal response obtained after treatment, reflecting the actual optical and topographical outcome induced by the lens design. Therefore, while BOZD acts as the adjustable input variable, it is the resulting PPRD that functionally predicts the treatment efficacy and the extent of axial length control. This clarification reinforces that clinical optimization in Ortho-k relies on tailoring lens parameters to achieve the desired PPRD configuration, which ultimately determines the retinal defocus profile and the biological response. In this study, a direct relationship was observed between BOZD and PPRD, suggesting that specific modifications of these parameters can be used to generate desirable optical aberration patterns that enhance myopia control efficacy.

The results obtained are consistent with previous research demonstrating a relationship between the amount of induced peripheral myopic defocus and the efficacy of myopia control [25,36]. Furthermore, studies have suggested that a reduced diameter of the power ring [BOZD] tends to generate a more effective defocus pattern on the peripheral retina, leading to improved myopia control [37].

This evidence is particularly relevant as it supports the notion that the specific design of Ortho-K lenses can enhance control of myopia progression, aligning with previous studies suggesting that managing axial length is a key strategy to prevent progressive myopia [17]. Additionally, significant differences were observed in scotopic pupil diameter between groups, but our results indicate that pupil size—whether photopic or scotopic—has no substantial impact on treatment efficacy, despite its potential to modify the distribution and effect of aberrations induced by Ortho-K lenses [26]. The observed relationship suggests that corneal shape parameters may play a more critical role than pupil size in predicting and influencing axial growth. In our findings only 12% of the children had a PPRD larger than the scotopic pupil itself, and no one at mesopic light had the PPRD larger than the pupil size. Therefore, our conclusions that pupil size plays a discrete role may be biased compared to other designs that induce larger PPRD due to the specific characteristics of the lens. The observed relationship suggests that corneal shape parameters may play a more critical role than pupil size in predicting and influencing axial growth.

While several group differences reached statistical significance, their clinical relevance warrants careful consideration. The absolute mean difference in axial elongation between groups was 0.07 mm per year. Although this difference was statistically significant (*p* = 0.008) and of moderate magnitude (Cohen’s d = 0.63), its immediate clinical impact appears modest. Translating axial length change into refractive terms is approximate and age-dependent; however, a commonly accepted estimate in pediatric myopia control research suggests that 0.10 mm of axial elongation corresponds to approximately 0.24–0.30 diopters of refractive change [38]. Accordingly, the observed 0.07 mm difference would equate to roughly 0.17–0.21 D per year. Although relatively small over a single year, a sustained effect of this magnitude could accumulate to a clinically meaningful reduction in myopia progression if maintained over time. The moderate effect size further indicates that the observed change is not trivial, reinforcing the potential benefit of smaller treatment-zone designs. Nonetheless, these results should be interpreted in the context of visual quality and higher-order aberration changes, as induced optical aberrations may counteract potential functional effects, such as contrast sensitivity, retinal image quality, and overall visual performance (visual acuity, clarity, or visual comfort). Future prospective studies with larger cohorts and extended follow-up are warranted to clarify the long-term clinical significance of these results.

This study provides additional evidence regarding the influence of higher-order aberrations [HOAs] [19] induced by Ortho-K treatment. A discrepancy between groups was evident for Vertical Coma, with the group having PPRD < 4.5 mm showing almost no change, whereas the group with PPRD ≥ 4.5 mm exhibited a significant increase of +0.084 ± 0.160. For horizontal coma, the group with PPRD < 4.5 mm showed a significant increase of +0.075 ± 0.153 microns (*p* = 0.004), whereas the group with PPRD ≥ 4.5 mm exhibited relevant changes in opposite sign with an increase of −0.058 ± 0.211 microns [*p* = 0.060]. This divergence suggests that lens BOZD design may selectively modulate different types of aberrations, potentially affecting the peripheral myopic defocus stimulus in distinct ways [39]. Previous studies [35] have confirmed that coma aberration increases after Ortho-K treatment, though most do not differentiate between vertical and horizontal coma, analyzing total coma instead. Other authors have not found these differences between groups of small versus large BOZD [24], possibly due to their methodology, which used anterior corneal wavefront aberrations calculated from topography maps rather than total ocular aberrations. From a clinical perspective, the variability in vertical coma observed in the higher PPRD group may reflect reduced post-treatment optical stability, particularly under conditions of pupil dilation [40]. The observed difference in horizontal and vertical coma aberrations may have distinct implications for the distribution of peripheral blur at the central or mid-peripheral retina and, therefore, for the effectiveness of myopic control. Recent research has identified a “sweet spot” in the mid-periphery (around 6–10 degrees eccentricity) as a key area for this growth regulation mechanism [32]. The significant increase in positive horizontal coma detected only in the group with PPRD < 4.5 mm could induce an asymmetric shift in the retinal image, increasing the growth-inhibiting signal in that area.

It is important to note that the present study evaluated only on-axis (central) ocular aberrations. Therefore, any interpretation regarding peripheral retinal mechanisms must be considered speculative within the context of our dataset. Although theories of myopia control frequently emphasize the role of mid-peripheral myopic defocus and peripheral image quality degradation, we did not directly measure peripheral wavefront aberrations or off-axis optical behavior. The references to peripheral mechanisms in this section are therefore intended solely as potential explanatory frameworks that may help contextualize the observed on-axis changes—particularly the differential behavior of horizontal and vertical coma [15]—but they should not be interpreted as direct evidence derived from the measurements collected. Future studies incorporating peripheral wavefront assessments or wide-field aberrometry would be required to confirm whether the on-axis aberration patterns identified here translate into specific mid-peripheral optical signals relevant to myopia control.

Both groups demonstrated significant increases in spherical aberration post-treatment (*p* < 0.001), with no significant differences between them. This result supports the hypothesis that spherical aberrations play a key role in modulating the peripheral retinal defocus signal, an aspect considered relevant for inhibiting axial growth [41].

Pupil size has been shown to play a relevant role, with better outcomes apparently observed when myopic defocus is partially within the pupillary area [42]. According to Chen et al. [23], pupil size plays a critical role in Ortho-K treatment, as the proportion of the pupil covered by the treatment zone (plus power ratio, PPR) influences the balance between myopic and hyperopic defocus. In this regard, a larger relative pupil size for the same BOZD increases the PPR, thereby optimizing myopic defocus on the retina. However, we did not find a significant relation between pupil size and AL in either group or across all participants. These findings indicate that axial elongation at one year is predominantly influenced by mean PPRD, whereas baseline refractive error and pupil size play a negligible role in predicting AL progression in this cohort.

This work also demonstrates a direct relationship between BOZD and PPRD, suggesting that specific modifications of these parameters could be employed to generate optical aberration patterns that optimize myopia control [26,29].

In summary, the results of this study suggest that a PPRD smaller than 4.5 mm may be more effective in reducing axial length growth in myopic children treated with Ortho-K, possibly due to its impact on horizontal coma aberrations.

Furthermore, a larger PPRD value was associated with greater ocular axial growth over one year of follow-up, which may have important clinical implications for myopia progression.

A methodological limitation of this study is that ocular aberrations were measured without cycloplegia, which is particularly relevant in pediatric patients, as active accommodation may influence both lower- and higher-order aberrations. Although procedures were implemented to minimize accommodative fluctuations, residual accommodation cannot be fully excluded, and therefore interpretation of aberration outcomes should be conservative. Beyond measurement-related limitations, the study sample size should also be considered. Based on a standard α = 0.05, β = 0.08, a common standard deviation of 0.15 mm, a meaningful change of 0.10 mm, and a projected 10% drop-out rate, an estimated 40 patients per group were required. While the final sample may be slightly underpowered, no participants withdrew from the study, and Type II errors are particularly relevant only when statistical significance is not achieved, which was not the case in this analysis.

Future prospective studies with larger cohorts and longer follow-up periods are warranted to elucidate the long-term clinical significance of these findings.

## 5. Conclusions

This study demonstrates that a smaller PPRD in Ortho-K treatment is significantly associated with reduced axial length growth and specific changes in higher-order aberrations in myopic children. A PPRD smaller than 4.5 mm was linked to a lower annual increase in axial length, suggesting greater potential for myopia control. These findings reinforce that it is the corneal PPRD—resulting from lens design adjustments—that predicts treatment efficacy, highlighting the importance of optimizing the corneal reshaping outcome rather than the nominal BOZD parameter itself.

## Figures and Tables

**Table 1:** Baseline Characteristics values and differences by the two groups.

	PPRD < 4.5 mm (*n* = 33)	PPRD ≥ 4.5 mm (*n* = 33)	Difference	*p*
Age (years)	13.39 ± 1.29	13.29 ± 1.50	0.11 ± 0.34	0.757 ^a)^
Sex (female/male)	8/25	19/14		
M Baseline (D)	−2.94 ± 1.24	−3.12 ± 1.52	0.18 ± 0.34	0.301 ^b)^
Pupil Photopic (mm)	4.34 ± 0.63	4.14 ± 0.53	0.20 ± 0.14	0.084 ^a)^
Pupil Scotopic (mm)	6.58 ± 0.80	5.95 ± 1.01	0.63 ± 0.22	0.003 *^a)^
Mean PPRD (mm)	4.06 ± 0.26	5.12 ± 0.39	−1.06 ± 0.08	<0.001 *^a)^
BOZD (mm)	5.01 ± 0.34	6.02 ± 0.50	−1.02 ± 0.11	<0.001 *^b)^
AL Baseline (mm)	24.52 ± 0.79	24.74 ± 0.99	−0.22 ± 0.22	0.158 ^a)^
AL post (mm)	24.60 ± 0.80	24.90 ± 1.00	−0.30 ± 0.22	0.092 ^a)^
AL Change at 1Y (mm)	0.08 ± 0.12	0.15 ± 0.10	−0.07 ± 0.03	0.008 *^a)^

Values are presented as mean ± standard deviation unless otherwise indicated. Primary comparisons between groups were evaluated using a Bonferroni-adjusted significance threshold of *p* < 0.025 (0.05/2). Significant results according to this corrected threshold are marked with *. ^a)^ Independent samples *t*-test (parametric); ^b)^ Mann–Whitney U test (non-parametric).

**Table 2:** Changes in higher-order aberrations, results, and differences between PPRD groups. Vertical Coma (VC), Horizontal Coma (HC), Coma total RMS (Coma RMS) and Spherical Aberration RMS (SA RMS).

		Pre ± SD (μm)	Post ± SD (μm)		Difference (Post-Pre)	*p*
VC	<4.5 mm	−0.013 ± 0.063	−0.015 ± 0.202		−0.002 ± 0.214	0.475 ^c)^
	≥4.5 mm	−0.006 ± 0.072	0.078 ± 0.161		+0.084 ± 0.160	0.003 *^c)^
	Diff	−0.007 ± 0.017, *p* = 0.340 ^a)^		−0.093 ± 0.045, *p* = 0.021 *^b)^		
HC	<4.5 mm	−0.004 ± 0.060	0.071 ± 0.153		+0.075 ± 0.153	0.004 *^c)^
	≥4.5 mm	0.005 ± 0.058	−0.053 ± 0.210		−0.058 ± 0.211	0.061 ^c)^
	Diff	−0.009 ± 0.014, *p* = 0.263 ^a)^		0.124 ± 0.045. *p* = 0.004 *^b)^		
Coma RMS	<4.5 mm	0.075 ± 0.042	0.211 ± 0.153		0.136 ± 0.159	<0.001 *^c)^
	≥4.5 mm	0.077 ± 0.050	0.195 ± 0.199		0.118 ± 0.218	0.002 *^c)^
	Diff	−0.001 ± 0.011, *p* = 0.454 ^a)^		0.017 ± 0.045, *p* = 0.353 ^b)^		
SA RMS	<4.5 mm	0.061 ± 0.021	0.195 ± 0.078		0.134 ± 0.080	<0.001 *^c)^
	≥4.5 mm	0.070 ± 0.022	0.198 ± 0.108		0.128 ± 0.112	<0.001 *^c)^
	Diff	−0.009 ± 0.005, *p* = 0.037 ^a)^		−0.004 ± 0.023, *p* = 0.440 ^b)^		

Values are presented as mean ± standard deviation unless otherwise indicated. Primary comparisons between groups were evaluated using a Bonferroni-adjusted significance threshold of *p* < 0.025 (0.05/2). Significant results according to this corrected threshold are marked with *. ^a)^ Independent samples *t*-test (parametric); ^b)^ Mann–Whitney U test (non-parametric); ^c)^ Wilcoxon signed-rank test (non-parametric).

## Data Availability

The original contributions presented in this study are included in the article. Further inquiries can be directed to the corresponding authors.

## Abbreviations

Ortho-K	Orthokeratology
PPRD	Plus Power Ring Diameter
D	Diopters
mm	Millimeters
AL	Axial length
HOAs	High-order aberrations
BOZD	Back Optic Zone Diameter
PPR	Plus Power Ratio
μm	Microns
